# Endothelial dysfunction correlates with decompression bubbles in rats

**DOI:** 10.1038/srep33390

**Published:** 2016-09-12

**Authors:** Kun Zhang, Dong Wang, Zhongxin Jiang, Xiaowei Ning, Peter Buzzacott, Weigang Xu

**Affiliations:** 1 Department of Diving and Hyperbaric Medicine, Faculty of Naval Medicine, the Second Military Medical University, Shanghai, China; 2 Department of Cadre Recuperation, Hangzhou Naval Sanatorium, Hangzhou, China; 3 School of Sports Science, Exercise and Health, the University of Western Australia, Perth, Australia

## Abstract

Previous studies have documented that decompression led to endothelial dysfunction with controversial results. This study aimed to clarify the relationship between endothelial dysfunction, bubble formation and decompression rate. Rats were subjected to simulated air dives with one of four decompression rates: one slow and three rapid. Bubble formation was detected ultrasonically following decompression for two hours, before measurement of endothelial related indices. Bubbles were found in only rapid-decompressed rats and the amount correlated with decompression rate with significant variability. Serum levels of ET-1, 6-keto-PGF1α, ICAM-1, VCAM-1 and MDA, lung Wet/Dry weight ratio and histological score increased, serum NO decreased following rapid decompression. Endothelial-dependent vasodilatation to Ach was reduced in pulmonary artery rings among rapid-decompressed rats. Near all the above changes correlated significantly with bubble amounts. The results suggest that bubbles may be the causative agent of decompression–induced endothelial damage and bubble amount is of clinical significance in assessing decompression stress. Furthermore, serum levels of ET-1 and MDA may serve as sensitive biomarkers with the capacity to indicate endothelial dysfunction and decompression stress following dives.

Bubble formation in tissues and circulating blood due to inadequate decompression is a causative factor in the pathogenesis of decompression sickness (DCS)^1^. Damage to vascular endothelial cells by decompression stress has been reported in a number of studies^2^,^3^,^4^,^5^,^6^,^7^,^8^,^9^. Animal experiments have demonstrated that simulated diving could cause endothelium-stripping and a reduction in endothelial-dependent vasorelaxation of pulmonary arteries in animals^2^,^3^,^4^,^7^. Circulating microparticles (MPs), which serve as sensitive markers of activation and dysfunction of endothelia, were also increased in humans and mice^5^,^6^,^8^,^9^. Incidence of DCS has been decreased by administration of short-acting nitric oxide (NO) donors and simvastatin, which have protective effects on endothelia in rats and pigs^10^,^11^,^12^. The above evidence strongly supports the hypothesis that endothelial cells are targets of DCS.

However, the etiology of endothelial injury after decompression is controversial. Some studies found that damage was caused by direct bubble-endothelia interaction, others suggested bubbles were not the primary causative agent of endothelial damage and showed that alternative factors such as increased formation of reactive oxygen species (ROS) caused by high oxygen partial pressure (ppO_2_) during diving compromised endothelial function^13^,^14^,^15^,^16^,^17^. It is generally acknowledged that circulating decompression bubbles mainly exist in the venous system, will be trapped in the pulmonary circulation and should have little effect upon the arterial system^18^,^19^. However, in healthy divers either single or repeated dives reduced the flow-mediated dilation (FMD) of the brachial artery^20^,^21^,^22^,^23^. Decompression was also reported to lead to measurable endothelial dysfunction even when no venous bubbles were detected in rats^24^. Although a correlation between bubble abundance and DCS has been shown, the relationship between bubble amount and endothelial damage remains uncertain^19^,^21^,^25^,^26^.

The aim of the present study was to clarify the relationship between bubble formation, decompression rate and endothelial dysfunction by observing changes in endothelial parameters following different decompression profiles in rats, and to screen possible biomarkers which may have potential clinical significance in assessing decompression injuries.

## Methods

### Animals

A total of 64 male rats (Sprague-Dawley strain) weighing 290–310 g were used for the experiments. The experiment protocol was approved by the Animal Ethics Committee of Second Military Medical University and the methods were carried out in accordance with the relevant guidelines, including any relevant details. Rats were housed in a controlled environment with a 12/12-h light/dark cycle, constant temperature (23 ± 1 °C) and relative humidity (54 ± 2%), with *ad libitum* access to a pelleted rodent diet and water.

### Grouping and treatment

**Figure 1:**
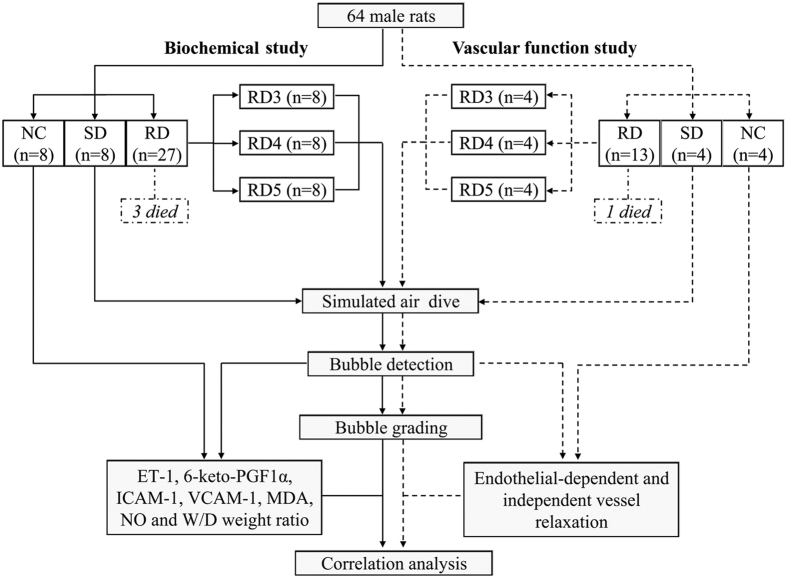
Flow chart describing the experimental design. NC, SD and RD denotes normal control, slow decompression and rapid decomression, respectively. RD3, RD4 and RD5 stands for decompression in 3, 4 and 5 min from a 7 ATA-90 min simulated air dive, respectively.

The rats were randomly divided into three groups: rapid decompression (RD), slow decompression (SD) and normal control (NC). The RD rats were further divided into 3 subgroups according to decompression rates. The SD and RD rats were subjected to a simulated air dive and bubbles flowing through the pulmonary artery were determined ultrasonically for analysis after surfacing. The NC rats were sham exposed (normobaric air) for the same length of time. Surviving rats in the SD and RD groups and the NC rats were anesthetized and sacrificed following bubble detection for measurement of endothelial related parameters. To avoid the possible interference on entothelial function by blood sampling, the study was separated into two parts: one for biochemical investigation including pulmonary edema and histological study, the other for pulmonary artery function. The experimental design and exact number of animals in each group are shown in Fig. 1.

### Simulated diving

The SD and RD rats were compressed with air to 7 absolute atmospheres (ATA) in 5 min and maintained for 90 min before decompression in a transparent hyperbaric rodent chamber (Type RDC150-300-6, SMMU, Shanghai, China). Compression was performed at an increasing rate from 1 ATA/min to 1.5 ATA/min to minimize middle ear squeeze in the animals. Decompression was carried out linearly to ambient pressure in 12 min (0.5 ATA/min) for SD rats and in 3, 4, 5 min for RD3, RD4 and RD5 subgroups (2.0 ATA/min, 1.5 ATA/min, 1.2 ATA/min), respectively.

### Bubble detection and grading

Immediately after surfacing, the SD and RD rats were anaesthetized with 10% chloral hydrate (3 mL/kg body weight) (Sinopharm Chemical Regent Co., Shanghai, China) intraperitoneally and were lain supine on a thermo-regulating pad (32 °C). The fur on the chest was removed. The cross-section at the root of the pulmonary artery was monitored for bubbles using an ultrahigh frequency (18 MHz) detector connected to an ultrasonic scanner (Mylab30cv, Esaote, Italy). All the manipulation before attaching the detector to the pulmonary artery was finished within 5 min, and detection was repeated at 5, 10, 20, 30, 45, 60, 90 and 120 min after decompression, each lasting for 60 s. Data were stored and played back in slow motion for analysis. The number of bubbles was scored according to the grading system described elsewhere^25^.

### Measurement of endothelial indices

Venous blood was drawn from the right ventricle under anesthesia. Blood was transfused into 2-ml Eppendorf tubes without any anticoagulation and placed in room temperature for 2 h. Then the samples were centrifuged at 1000 g at 4 °C for 20 min. The supernatant was stored at −80 °C until determination. The serum levels of endothelin-1 (ET-1), 6-keto-PGF1α, intercellular cell adhesion molecule-1 (ICAM-1) and vascular cell adhesion molecule-1 (VCAM-1) were assayed by ELISA (Elabscience Biotechnology, Wuhan, China). Levels of nitric oxide (NO) and malondialdehyde (MDA) were detected by chemical colorimetry using commercial assay kits (Jiancheng Bioengineering Institute, Nanjing, China). All assays were performed following the respective manufacturer’s instructions.

Lung wet-dry weight and histology were also measured. The weight of the lung tissue was determined from a less than 1 g section of the left lung. The tissue was weighed (wet weight), incubated at 120 °C for 3 days and then weighed again (dry weight). Lung W/D weight ratio was used to estimate the severity of pulmonary edema. Samples from right upper lung were paraffin embedded by conventional procedures, sectioned into slices of 5-μm thickness. Hematoxylin and eosin (HE) staining was conducted and morphological changes were observed under a microscope. Ten different areas were selected randomly from each specimen and examined using a computer image analysis system (Smart Scape, Furi Science & Technology Co., ltd, Shanghai, China). Histological scoring was scaled as described previously^12^. Briefly, score 0 represents normal histology; score 1 represents slight leukocyte infiltration and capillary congestion; score 2 represents mild leukocyte infiltration, perivascular edema, partial damage of pulmonary structures, and hemorrhage; score 3 represents intense leukocyte infiltration and destruction of pulmonary structures.

### Pulmonary artery tension measurement

The chest of each rat in the pulmonary artery analysis group was opened under anesthesia, the heart and lungs were harvested and immediately immersed in cold Krebs-Henseleit solution [K-H solution, composition (mM): NaCl, 118.0; KCl, 4.7; MgSO_4_, 1.2; KH_2_PO_4_, 1.2; CaCl_2_, 2.5; NaHCO_3_, 25.0; and glucose, 11.1; pH 7.4]. The second branch of the right pulmonary artery was gently dissected and the connective tissue and fat carefully removed. The vessels were cut into 3 mm long rings, mounted on two L-shaped metal prongs and lowered into a temperature-controlled (37 °C) tissue bath containing K-H solution. The solution was aerated continuously with a mixture of 95% O_2_ and 5% CO_2_. Changes in tension were recorded by isometric transducers connected to a data acquisition system (ALCB10, MPA-2000, Alcott Biotech, China). The solution was changed every 15 min. Rings were stretched progressively to a basal tension of 0.5 g and allowed to equilibrate for 1 h. The contractile capacity of each vessel segment was measured by exposure to a potassium-rich (60 mM) K-Krebs buffer solution that had the same composition as the Na-Krebs buffer solution except that some of the NaCl was replaced by an equimolar concentration of KCl. For relaxation measurement the rings were pre-contracted with phenylephrine (PE) at a concentration of 10^−5 ^M. When the PE-induced contraction reached a plateau level, acetylcholine (ACh, 10^−9^‒10^−5 ^M) was added in cumulative concentration. The resultant relaxation response indicated how severely the endothelial layer was damaged by bubbles. The performance of the smooth muscle layer was examined with cumulative doses of sodium nitroprusside (SNP, 10^−10^‒10^−5 ^M). Changes in tension induced by ACh or SNP were expressed as percentages of the initial contraction induced by PE.

### Statistical analysis

Unless otherwise stated, all data are presented as mean ± SD. One-way ANOVA followed by Student Newman–Keuls tests or post hoc Dunnett’s tests were used for multiple comparisons between means. Lung histological scores between RD and SD rats and the difference in dependent correlations were compared using Student’s *t*-test. Relaxation responses of arterial rings are expressed as percentage reversal of the phenylephrine contraction. Pearson correlation and Spearman correlation were used for correlation analysis between endothelial indices and bubble counts and between endothelial indices and decompression rate, respectively. The threshold for significance was accepted at *P *< 0.05.

## Results

### Bubble formation

**Figure 2:**
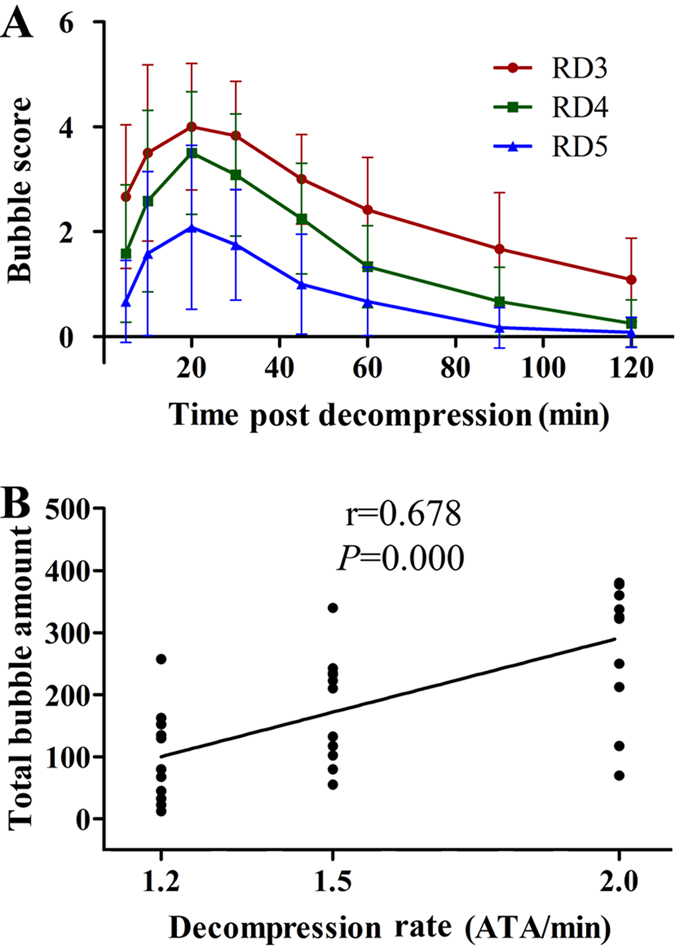
Bubble formation in rats decompressed with different rates (**A**) and correlation between bubble formation and decompression rate (**B**). Rats were exposed to 7 ATA air for 90 min and decompressed to atmospheric pressure in 3, 4, or 5 min (RD3, RD4, and RD5). Bubbles were detected and scored at 8 time points following decompression. Total bubble count for each rat was calculated as the area under the curve. Error bars are standard deviation to the mean. n = 12 for each of the RD group. Some dots overlap in Panel B.

Immediately after decompression, bubble detection was performed at the root of the pulmonary artery. Gas bubbles were seen as moving bright spots in ultrasound images of the pulmonary artery in RD rats only. The relative bubble count increased gradually after decompression, reaching a maximum at around 20 min in all subgroups. Faster decompressed rats showed greater total bubble count represented by the area under the curve, 286.5 ± 103.1, 184.4 ± 85.0, 92.5 ± 75.8 for RD3, RD4 and RD5, respectively (*P *< 0.01 for RD3 *vs.* RD4 or RD5, *P *< 0.05 for RD4 vs. RD5, Fig. 2A). Linear regression revealed a significant positive relationship between decompression rate and bubble formation (Fig. 2B). However, bubble counts showed wide dispersion even among rats decompressed at the same rate, with coefficients of variation (CV) of 34%, 44% and 79% for RD3, RD4 and RD5 respectively.

### Endothelial indices

**Figure 3:**
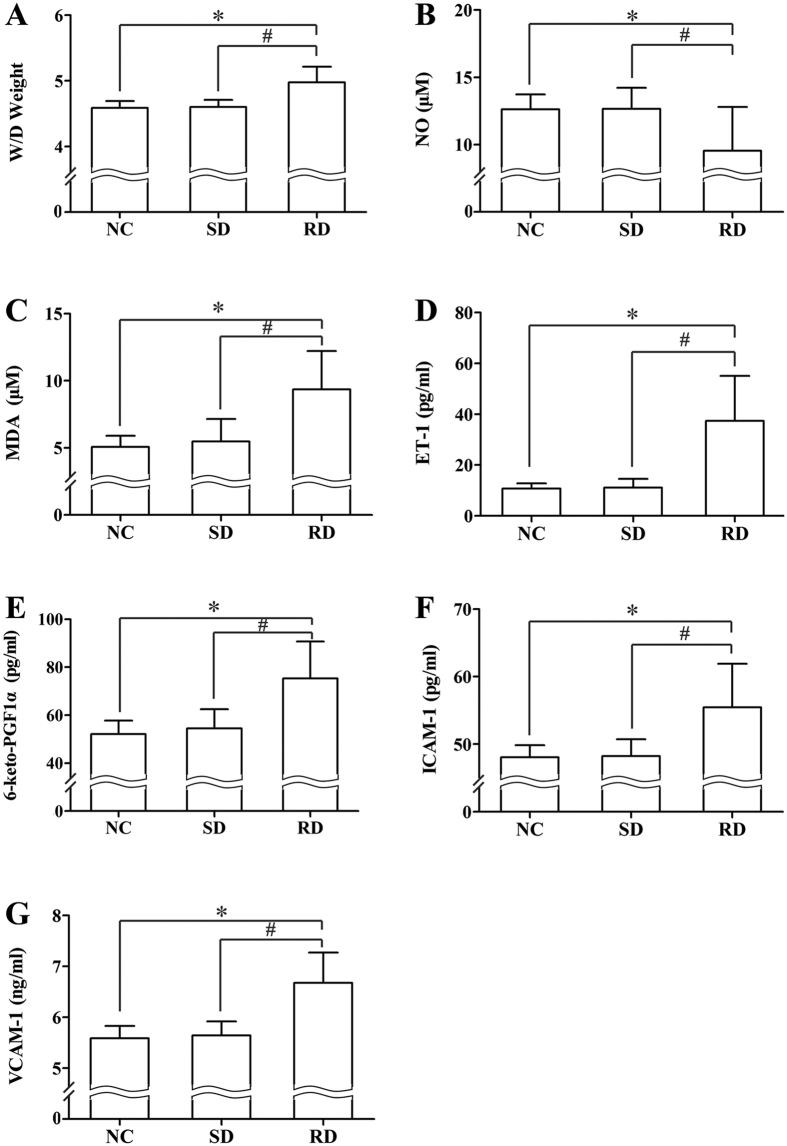
Decompression induced changes in endothelial biomarkers. All parameters were determined at 2 h after decompression from a simulated air dive (7 ATA-90 min) in 12 min (SD) or 3, 4 or 5 min (RD). n = 8 for NC and SD group, n = 24 for SD group, which included the 3 subgroups (n = 8 per subgroup). Error bars are standard deviation to the mean. *P < 0.01 RD *vs.* NC rats respectively, ^#^*P *< 0.05 RD *vs.* SD rats.

**Figure 4:**
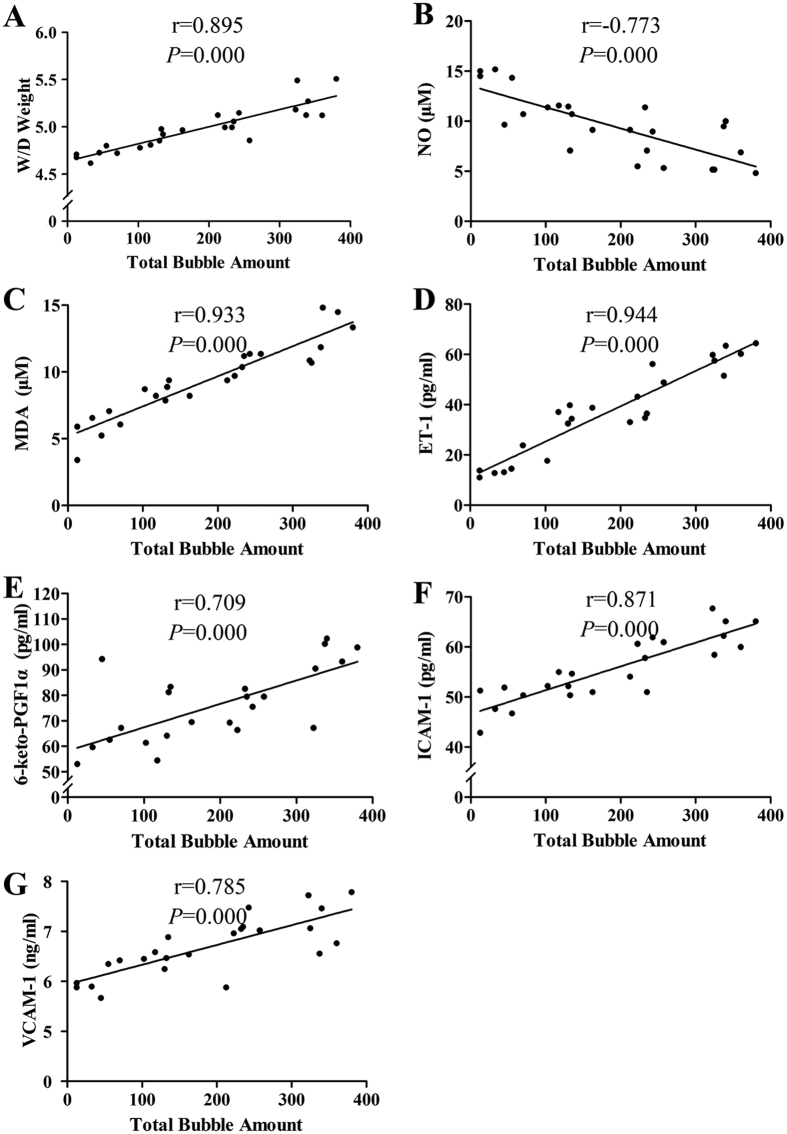
Correlation analysis between the endothelial damage and total bubble amount. Endothelial indices and bubbles were detected in rats rapidly decompressed from a simulated dive (7 ATA-90 min) in 3, 4 and 5 min. Total bubble count is represented by the area under the curve showed in Fig. 2A. n = 24 and some dots overlap.

**Figure 5:**
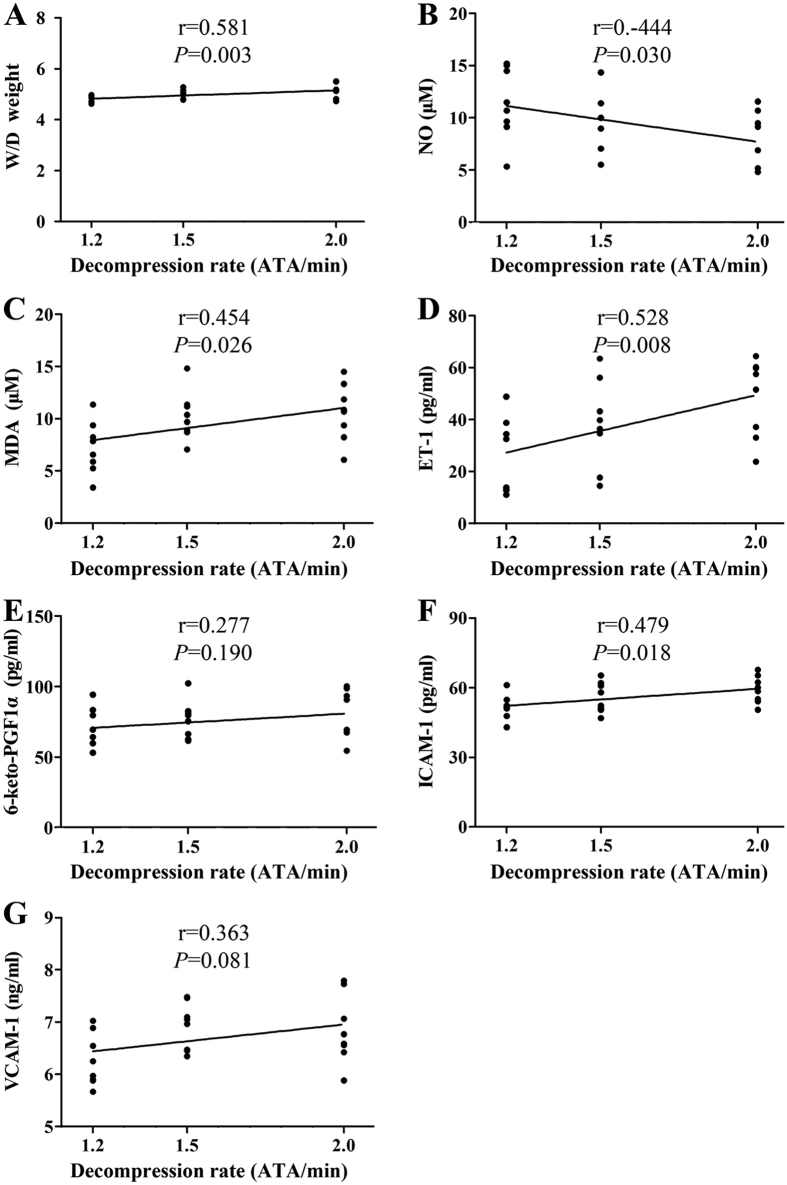
Correlation between endothelial biomarkers and decompression rate. Rats were exposed to 7 ATA air for 90 min and decompressed to atmospheric pressure in rate of 1.2, 1.5, or 2.0 ATA/min (RD5, RD4, and RD3) and the endothelial indices were determined 2 h after decompress. n = 8 for each decompression rate and some dots overlap.

No difference was found between the NC and SD rats. Rapid decompression induced a significant increase in serum ET-1, 6-keto-PGF1α, ICAM-1, VCAM-1 and MDA levels, lung Wet/Dry weight ratio, and a significant decrease in serum NO level (Fig. 3, *P *< 0.01 RD vs. SD or NC group). Significant decreases in NO correlated negatively with bubble counts (*P *< 0.01) and decompression rates (*P *< 0.05), while all the increases of the other parameters correlated positively with bubble counts (*P *< 0.01) and decompression rates (*P *< 0.05) with exception of 6-keto-PGF1α and VCAM-1, which showed no correlation with decompression rate (Figs 4 and 5).

### Lung morphological changes

**Figure 6:**
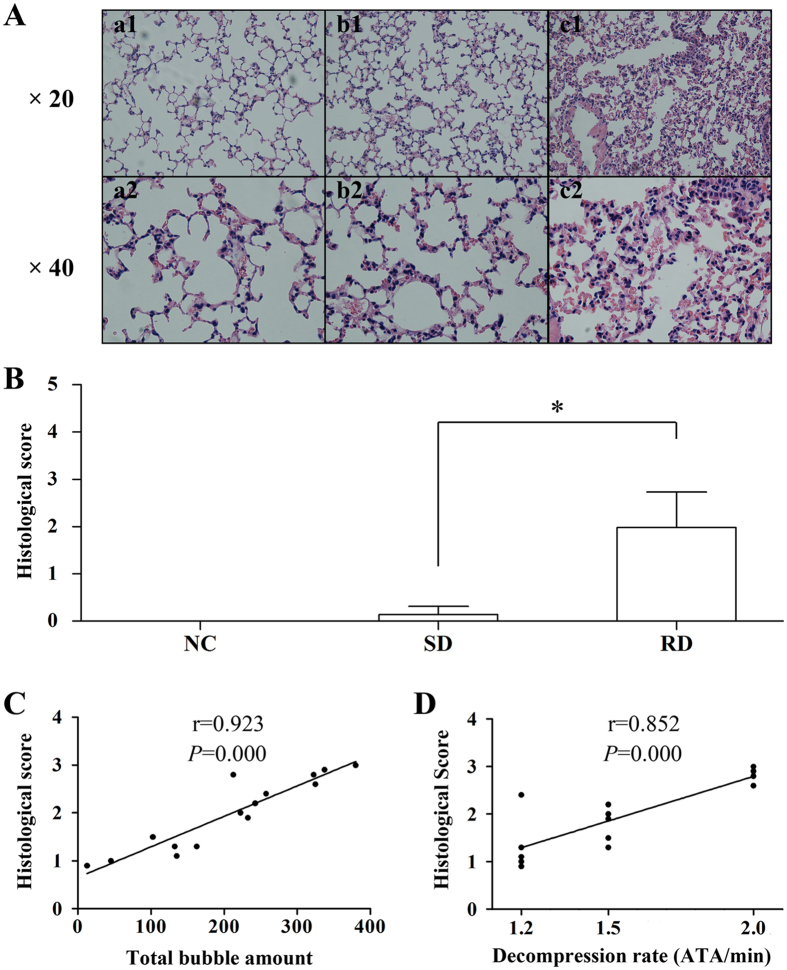
Photomicrographs and histological score of lung biopsies and correlation with decompression rate and bubble counts. Tissues were sampled from normal control (NC, (**A**)) slow decompressed (SD, (**B**)) and rapid decompressed (RD, (**C**)) rats. The higher the histological score, the greater severity of the injury. One of the dots is overlapped in panel D. Error bars are standard deviation to the mean. *P < 0.001 RD *vs.* SD, n = 5 in NC or SD group, n = 15 in RD group.

Rapid decompression caused injuries including edema, capillary expansion and congestion, hemorrhage, leukocyte infiltration, disruption of the structure (Fig. 6A) and RD3 rats scored highest (Fig. 6B). The score in RD lungs correlated positively with bubble counts and decompression rates (*P *< 0.01) (Fig. 6C,D).

### Changes in pulmonary artery tension

**Figure 7:**
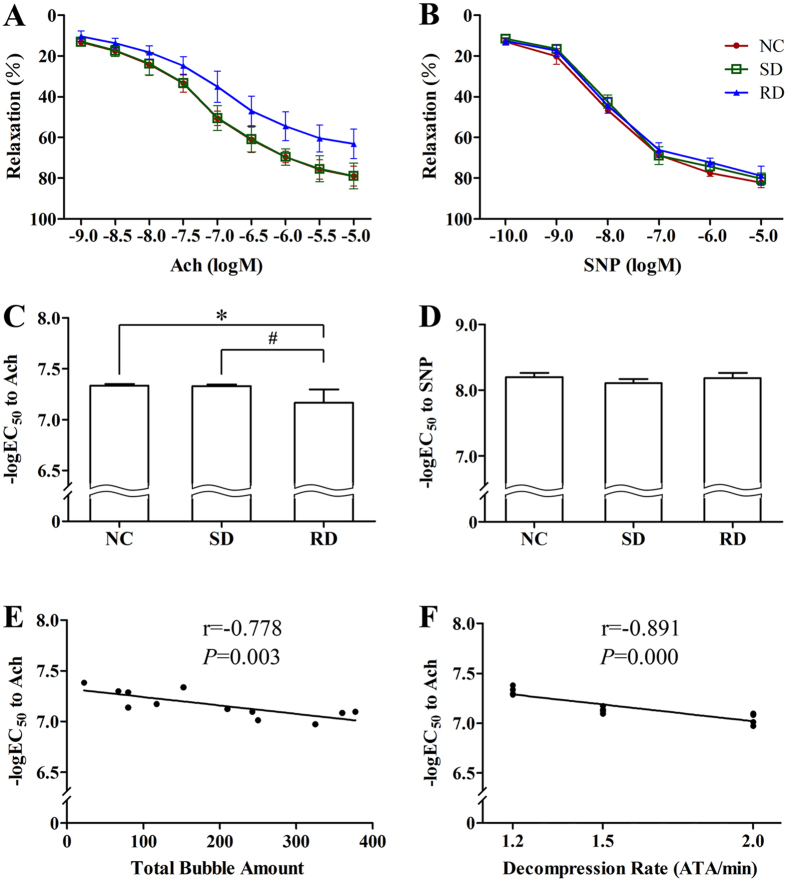
Concentration-response relaxation in isolated rat pulmonary artery rings and the correlation with decompression rate or bubble count. The Ach and SNP induced relaxation (**A**,**B**) and the EC50 values (**C**,**D**) were determined on isolated rings from the second branch of the pulmonary arteries in rats rapid decompressed (RD) or slow decompressed (SD) from a simulated air dive to 7 ATA for 90 min. The correlation between -logEC_50_ with decompression rate (**E**) or total bubble count (**F**). NC stands for normal control. The EC50 to Ach or SNP were the concentrations that produced 50% relative responses. Error bars are standard deviation to the mean. *P < 0.01 RD *vs.* NC rings, ^#^*P* < 0.05 RD *vs.* SD rings. n = 4 in NC or SD group, n = 12 in RD group. Some dots in Panel F overlap.

Concentration-response relaxation curves were obtained by adding Ach (10^−9^–10^−5 ^mol/L) or SNP (10^−10^–10^−5 ^mol/L) cumulatively (Fig. 7A,B). The percentages of relaxation of artery rings differed when stimulated at different concentrations. The concentration at which 50% of the response (EC_50_) to Ach was observed increased significantly in RD rings compared with NC or SD rings (*P *< 0.01, Fig. 7C). There was no difference in the EC_50_ to SNP among the three groups (*P *> 0.05, Fig. 7D). The -logEC_50_ to Ach correlated negatively with bubble counts (Fig. 7E) or decompression rate (Fig. 7F), indicating positive correlations among endothelial dysfunction, bubble counts and decompression rates.

## Discussion

DCS is a potential problem for a growing population of professional and recreational divers^1^. There is a common agreement that bubble formation is associated with DCS^1^,^27^. Evidence points to vascular endothelial cells as a target for damage by decompression stress^2^,^3^,^4^,^5^,^6^,^7^,^8^,^9^,^28^. However, the cause of endothelial injury after decompression is controversial^13^,^15^,^16^. Elucidating the correlation between bubble amount, decompression rate and endothelial dysfunction will help to understand the etiology of endothelial damage after decompression and identify reliable methods for assessing decompression stress.

To induce different amounts of decompression bubbles in rats, three different decompression rates were adopted. According to our preliminary study, for a 7 ATA-90 min simulated air dive, 12 min decompression duration would not induce observable bubble formation for rats weighing around 300 g; while 3–5 min decompression causes a variety of DCS symptoms (further verified by the present results)^12^,^29^. In the three RD subgroups, faster decompression produced greater amounts of bubbles. However, bubble formation showed significant variability even among rats with low weight variation and with decompression at the same rate, with coefficient of variation ranging from 34% to 79% between the three subgroups. This finding coincides with observations that individual variation in DCS susceptibility exists in divers^19^,^30^.

A series of endothelia related indices were measured in the present study. Among these determined parameters, the lung Wet/Dry weight ratio is an indicator of lung microvascular permeability; VCAM-1 and ICAM-1 are sensitive biomarkers with the capacity to reflect endothelial damage directly; NO, ET-1 and 6-keto-PGF1α are vasoactive substances secreted by endothelial cells; lung morphology reflects the general injuries caused by bubbles; and *in vitro* vasodilatation determination is considered to be a reliable method for evaluating endothelial and smooth muscle function. Rapid decompression induced significant changes in all these parameters. Slow decompression did not induce detectable bubbles and none of the determined indices changed, compared with normal controls, which suggests that neither the high ppO_2_ nor hyperbaric exposure *per se* exerted any discernable effects in this scenario. Increased ppO_2_ during diving has been proposed as a cause of endothelial dysfunction^16^. Linear regression revealed that all observed changes correlated well with bubble amounts in a dose-dependent manner. This is in accordance with previous studies which found reduction of endothelial-dependent vasodilation was related to the amount of bubbles^2^,^4^. These findings suggest that intravascular bubbles may be the causative agents of endothelial damage following diving decompression, and bubble score may be sensitive to predict decompression-induced endothelial dysfunction. Among the serum parameters measured in this study, ET-1 and MDA presented better sensitivity and reliability as demonstrated in the relatively large changes and high correlation shown in Figs 2 and 3. These serum indices could be valuable biomarkers for decompression stress but it remains to be tested if they are equally sensitive in other species and/or other dive profiles. Further studies in a swine DCS model and divers are underway. While 7 out of the 9 measured biomarker levels correlated with decompression rate, the mean correlation coefficient was not as high as the mean correlation between the indices and bubbles (0.552 ± 0.207 *vs.* 0.846 ± 0.086). As bubbles cause endothelial injury, the result also reflects the significant variability of bubble formation at the same decompression rate. Accordingly, we recommend future rat research incorporate bubble measurement into the study design, rather than just vary the rate of decompression and compare groups based on dive profile instead of between bubble score outcomes.

During inadequate decompression from a diving or hyperbaric exposure, bubbles will form in supersaturated tissue, converge into the venous system, and move with blood flow to the right heart and thence the pulmonary artery for sequestration in the lung vasculature. Decompression bubbles therefore mainly exist in the venous system^18^,^19^. In this study, no bubbles were found in the left heart and aorta. However, the present results could not determine whether injuries to endothelia were only from the venous system or from both sides. Because tiny moving microbubbles cannot be detected by current ultrasound techniques, the possibility remains that bubbles could have existed in the arterial system. In human divers, even large venous bubbles do cross to systemic arteries via right-to-left shunts including patent foramen ovale^20^ and, although bubble detection was not performed, insults to arterial smooth muscle have been reported in a rat DCS model while endothelial function remained unchanged^31^,^32^.

Circulating bubbles can act on the endothelial lining of vessels directly or via increased shear stress, resulting in perturbations, activation or even stripping of endothelial cells^25^. As with foreign material, bubbles can initiate biochemical cascades including activation of coagulation, platelet aggregation, inflammatory responses, and lead ultimately to endothelial damage^13^,^33^. No matter how bubbles injure endothelia, the indices measured in this study herald endothelial activation and dysfunction, and are sensitive markers of decompression stress, especially for the assessment of divers with subclinical manifestations. Furthermore, some substances released from injured endothelia will initiate further endothelial injury at remote sites^13^,^20^, making the etiology of this protean disease more complicated. This may also be a possible cause of arterial dysfunction in divers who have not obviously violated decompression rules^8^. This possibility deserves further study.

In this study, the relaxation of pulmonary artery rings from rapidly decompressed rats remained unchanged with SNP (a vasodilator acting directly on smooth muscles) but was hampered with Ach (a vasodilator acting via endothelia), indicating that vascular smooth muscle was not affected (Fig. 6). Similar findings have been previously reported^3^,^4^. However, a study on divers showed both endothelium dependent and independent vascular function were altered after a single air scuba dive, and vascular smooth muscle was postulated to be involved^21^. Another study on rats showed that vascular smooth muscle function was progressively impaired with increased decompression stress with no modification of endothelium-dependent vasorelaxation^31^. The different amount and size of bubbles formed, type of vessels and other predisposing factors could offer possible explanation^4^,^34^.

In conclusion, the present study suggests that endothelial dysfunction following decompression is mainly caused by bubbles in a linearly correlated relationship. The results further support the notion that bubble amount is a sensitive marker of decompression stress. Some of the endothelial indices together with bubble count may serve as sensitive or specific markers with the capacity to reflect decompression stress. Further studies are warranted to reveal the mechanisms of decompression induced bubbles affecting endothelial integrity and function.

## Additional Information

**How to cite this article**: Zhang, K. *et al*. Endothelial dysfunction correlates with decompression bubbles in rats. *Sci. Rep.* **6**, 33390; doi: 10.1038/srep33390 (2016).
